# Trends in smokeless tobacco use in the us workforce: 1987-2005

**DOI:** 10.1186/1617-9625-9-6

**Published:** 2011-06-01

**Authors:** Noella A Dietz, David J Lee, Lora E Fleming, William G LeBlanc, Kathryn E McCollister, Kristopher L Arheart, Evelyn P Davila, Alberto J Caban-Martinez

**Affiliations:** 1 University of Miami Miller School of Medicine, Sylvester Comprehensive Cancer Center, Department of Epidemiology & Public Health, 1120 NW 14 Street, 15 Floor C202, Miami, Florida 33136, USA; 2 University of Miami Miller School of Medicine, Sylvester Comprehensive Cancer Center, Department of Epidemiology & Public Health, 1120 NW 14 Street, 10 Floor, Miami, Florida 33136, USA

## Abstract

The primary aim was to examine whether increasing workplace smoking restrictions have led to an increase in smokeless tobacco use among US workers. Smokeless tobacco exposure increases the risk of oral cavity, esophageal, and pancreatic cancers, and stroke. The prevalence of smokeless tobacco use decreased from 1987-2000, except among men 25-44. While smokeless tobacco use has declined in the general population, it may be that the prevalence of smokeless tobacco use has increased among workers due to workplace smoking restrictions, which have been shown to have increased over the years. Using the most current nationally representative National Health Interview Survey (NHIS) data, we examined whether increasing workplace smoking restrictions have led to an increase in smokeless tobacco use among US workers (n = 125,838). There were no significant changes in smokeless tobacco use prevalence from 1987-2005 (pooled prevalence = 3.53%); rates also were lower in smoke free workplaces. Worker groups with high rates of smokeless tobacco use included farm workers (10.51%) and blue collar workers (7.26%). Results indicate that smokeless tobacco prevention strategies targeting particular worker groups are warranted.

## Background

Smokeless tobacco exposure increases the risk of oral cavity, esophageal, lung, and pancreatic cancers,[1-3] and stroke [4]. A number of carcinogens are present in smokeless tobacco products and users have higher urinary levels of known carcinogens, including 4-(methylnitrosamino)-1-(3-pyridyl)-1-butanone (NNK) and N'-nitrosonornicotine (NNN), than non-users [3,5]. Further, research shows comparable NNK exposures in smokeless tobacco users and smokers [5]. Smokeless tobacco use is higher in men, young adults, individuals living in non-metropolitan areas, whites, individuals with lower education levels, and people living in the Southern or Western United States [6,7]. Overall, the prevalence of smokeless tobacco use decreased from 1987-2000, except among men 25-44 [6,7]. Further, from 2002-2007, the prevalence of smokeless tobacco use, in general, among individuals 12 or older remained fairly stable [8]. While smokeless tobacco use declined and then stabilized in the general population, it may be that the prevalence of smokeless tobacco use has increased among workers due to workplace smoking restrictions, which have been shown to have increased over the years [9]. In 1999, approximately 70% of US workplaces had a workplace smoking restriction [10]. This figure increased to 77% by 2003, with some variation by occupation category [11]. There may be increases in smokeless tobacco use among workers as tobacco companies increase their advertising promoting smokeless tobacco products as an alternative to quitting smoking or as a way to cope with smoking restrictions [12-15]. In the present study, we examined the relationship between occupational status, the presence of workplace smoking restrictions, and smokeless tobacco use among a nationally representative sample of US adults.

## Methods

The most current data specifically asking respondents about their smokeless tobacco use from the 1987-2005 National Health Interview Survey (NHIS), an annual population-based survey of the entire US civilian population, were analyzed with adjustment for survey design [16]. Employed respondents 18 years of age and older reported their occupation for the one to two weeks prior to interview and were grouped into detailed occupational group classifications [17]. Smokeless tobacco use was assessed in years 1987, 1991-1994, 1998, 2000, and 2005 (n = 125,838). The NHIS smokeless tobacco use questions varied slightly across these survey years. From 1987-1994, there were two questions on smokeless tobacco: "Do you use chewing tobacco now" and "Do you use snuff now," with responses categorically coded (yes to either question = 1; no to both questions = 0). From 1998 through 2005, the NHIS again asked respondents two questions on smokeless tobacco, "do you use snuff tobacco every day, some days, or not at all" as well as "do you use chewing tobacco every day, some days, or not at all." Responses were dummy coded (1 = every day or some days; 0 = no). Smokeless tobacco questions were not included in later survey questionnaires.

We created four occupational groups based on the 1980 US Census Codes, which are often used by the National Center for Health Statistics. Occupational categories included: White Collar (census code 003-389), Blue Collar (503-889), Service Work (403-469), and Farm Work (473-499) [18]. Workers also were asked if there were any smoking restrictions at their workplace. A weighted linear regression model was fitted to the annual design-adjusted rates within occupational groups to assess smokeless tobacco use trends. Smokeless tobacco prevalence rates pooled across survey years for all workers were estimated, with appropriate adjustment for sampling weights [19] as well as for workers covered and not covered by workplace smoking restrictions. Analyses were completed using SUDAAN software, with adjustments for design [19].

## Results

**Figure 1:**
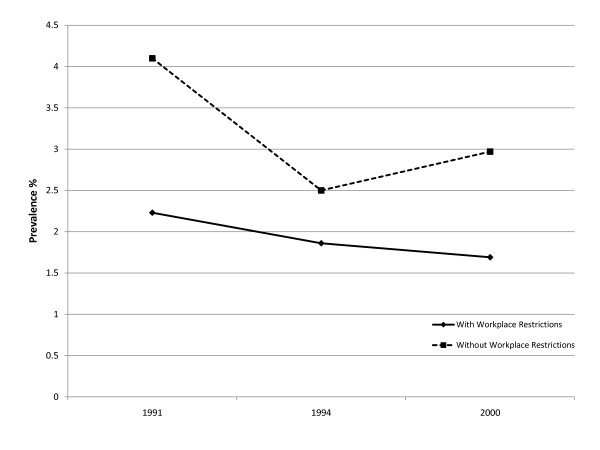
**Trends in Smokeless Tobacco Use: With and Without Workplace Restrictions**.

There were 125,838 survey participants representing an estimated annually employed 103 million US workers 18 years of age and older from 1987-2005 (Table 1). During the study period, there were an estimated 3.6 million workers or 3.53% of all US workers who reported smokeless tobacco use. Overall, there were no statistically significant upward or downward trends in smokeless tobacco rates among all US workers (see Figure 1). However, when we examined the prevalence rates of smokeless tobacco use between worker categories, we found significant differences with higher pooled rates for farm workers (10.51%) and blue collar workers (7.26%) relative to service (2.37%) and white collar (1.96%) workers.

**Table 1:** Average Annual Pooled Prevalence of Smokeless Tobacco Use in the United States by Occupation: The 1987, 1991-1994, 1998, 2000, and 2005 National Health Interview Surveys

					Workplace Smoking Restrictions Present‡	
					**Yes**	**No**

**Occupation**	**NHIS** **Pooled** **Sample** **Size**	**US** **Worker** **Population** **Estimate**	**US** **Worker** **Population** **Estimate of** **Smokeless** **Tobacco** **Use**	**Percent and** **[95% CI^†^]** **Using** **Smokeless** **Tobacco**	**Percent and** **[95% CI^†^]** **Using** **Smokeless** **Tobacco**	**Percent and** **[95% CI^†^]** **Using** **Smokeless** **Tobacco**

**All Workers**	125,838	103,073,889	3,635,403	**3.53** **[3.34-3.72]**	**1.89** **[1.70-2.11]**	**3.27** **[2.78-3.85]**
**White Collar**	75,392	61,229,033	1,198,169	**1.96** **[1.82-2.11]**	**1.16** **[0.99-1.36]**	**1.23** **[0.84-1.81]**

**Service**	18,062	13,893,418	328,908	**2.37** **[2.07-2.70]**	**1.73** **[1.22-2.46]**	**1.72** **[1.05-2.81]**

**Farm**	3,002	2,462,646	258,945	**10.51** **[9.19-12.00]**	**6.54** **[3.05-13.46]**	**9.36** **[5.29-16.03]**

**Blue Collar**	29,382	25,488,793	1,849,381	**7.26** **[6.79-7.75]**	**4.87** **[4.17-5.68]**	**6.93** **[5.75-8.34]**

^†^95% Confidence Interval

‡Respondents were asked this only in years 1991, 1994, and 2000 (n = 37,546)

When we examined smoking restrictions at the workplace, overall we found that workers who were employed in occupations where smoking was restricted had lower rates of smokeless tobacco use compared to workers who could smoke at work. Farm workers without smoking restrictions at work were more likely to use smokeless tobacco (9.36%) than farm workers with smoking restrictions (6.54%). The worker group least likely to use smokeless tobacco products was white collar workers with and without smoking restrictions (1.16% and 1.23%, respectively).

The prevalence of smokeless tobacco use by occupation also was evaluated by gender and ethnicity/race, with men reporting higher smokeless tobacco use than women, particularly men in farming occupations or white men in blue collar occupations (Table 2). In general, women reported the least amount of smokeless tobacco use, especially women in white collar occupations.

**Table 2:** Average Annual Pooled Prevalence of Smokeless Tobacco Use in the United States by Occupation and by Gender and Ethnicity/Race: The 1987, 1991-1994, 1998, 2000, and 2005 National Health Interview Surveys

Occupation by Gender and Ethnicity/Race	NHIS Pooled Sample Size	US Worker Population Estimate of Smokeless Tobacco Use	Percent and [95% CI^†^] Using Smokeless Tobacco
**White Collar**			
**White Men**	26,563	1,124,494	4.68 [4.35- 5.04]
**Black Men**	2,309	22,078	1.12 [0.59- 2.12]
**Other Men**	1,499	20,729	1.47 [0.90- 2.39]
**White Women**	37,546	27,496	0.09 [0.06- 0.14]
**Black Women**	5,679	2,228	0.06 [0.02- 0.18]
**Other Women**	1,796	1,144	0.08 [0.02- 0.40]

**Service**			
**White Men**	4,760	259,559	6.07 [5.21- 7.05]
**Black Men**	1,188	22,689	2.28 [1.28- 4.02]
**Other Men**	376	8,463	2.45 [1.26- 4.62]
**White Women**	8,446	13,026	0.21 [0.12- 0.36]
**Black Women**	2,721	22,882	1.52 [0.96- 2.39]
**Other Women**	569	2,289	0.54 [0.12- 2.36]

**Farm**			
**White Men**	2,169	236,256	12.70 [11.01- 14.61]
**Black Men**	149	14,655	14.65 [8.55- 23.97]
**Other Men**	109	4,503	5.66 [2.10- 14.37]
**White Women**	534	2,339	0.59 [0.12- 2.75]
**Black Women**	23	1,192	9.01 [1.28- 43.08]
**Other Women**	18	0	0.00 [0.03- 1.52]

**Blue Collar**			
**White Men**	19,385	1,716,170	9.59 [8.95- 10.26]
**Black Men**	2,761	67,867	2.97 [2.20- 4.01]
**Other Men**	990	31,144	3.50 [2.18- 5.56]
**White Women**	4,668	12,241	0.35 [0.23- 0.54]
**Black Women**	1,233	21,394	3.07 [1.81- 5.17]
**Other Women**	345	566	0.21 [1.03- 1.52]

^†^95% Confidence Interval

## Discussion

Smokeless tobacco products contain over 30 carcinogens and smokeless tobacco users have similar exposures to the tobacco specific carcinogens NNK and NNN as smokers [3,5]. Animal models testing the carcinogenic effects of smokeless tobacco show NNK to be the leading carcinogen in creating tumors in the lung, pancreas, nasal mucosa, and liver of rats [5]. Individuals exposed to smokeless tobacco have an increased risk of oral cavity, esophageal, lung, and pancreatic cancers [1-3], as well as stroke [4]. In this study, we examined the prevalence of smokeless tobacco use among US workers and determined if the presence of workplace smoking restrictions was associated with higher smokeless tobacco use. We found that most US workers did not use smokeless tobacco and that prevalence of smokeless tobacco rates was relatively stable throughout the study period. However, there were large differences in the use of smokeless tobacco across worker groups, with notably high rates of use in black and white male farm workers, white male blue collar workers, and white male service workers.

We did not find any evidence that workers exposed to workplace smoking restrictions were more likely to use smokeless tobacco products, despite industry efforts to promote smokeless tobacco as a harm reduction product or replacement product for cigarettes [12-15,20]. In fact, smokeless tobacco use was lower in workplaces with smoking restrictions, raising the possibility that the continued aggressive pursuit of the passage of clean indoor air legislation also will serve to lower, not increase, smokeless tobacco use. Past research has shown that smoke free workplace policies have been associated with decreases in tobacco consumption as well as a decrease in overall prevalence [21]. Moreover, population-based evidence has shown that smokers who have household smoking bans are more likely to have a successful quit attempt than smokers who do not [22-24]. Thus, as well as reducing the exposure of others to secondhand smoke, restrictions on places where smoking is allowed appears to increase the likelihood and success of a smoker making a quit attempt.

While the causal mechanisms enabling such a reduction are not known at the present time, it is possible that the process of the "denormalization" of smoking behavior via clean indoor air restrictions might also extend to smokeless forms of tobacco use [25]. Simply put, if individuals in their daily lives do not engage nor see others engaging in tobacco use, they are less likely to deviate from the normative environment surrounding them and use tobacco products.

Two limitations of the study should be addressed. First, it is a cross-sectional design using self-reported smokeless tobacco data; individual changes in tobacco behavior cannot be determined with this design. However, repeated cross-sectional surveys can track prevalence and estimates of use. Secondly, the smokeless tobacco use behavior questions were not asked for each survey year; rather, they were only asked for certain survey years and the last round of smokeless tobacco questions were asked in 2005. A more appropriate survey design would have been to ask the questions in each survey year through to the present, as smokeless tobacco products are still available for sale. While there were a couple of study limitations, the NHIS is a population-based survey of the entire US civilian population, a major strength of this study. In sum, smokeless tobacco prevention strategies specifically targeting farm workers and blue collar groups are warranted, particularly in light of their relatively high rates of use in the US workforce.

### What this paper adds

Previous work on smokeless tobacco use shows a decline in the general population, but it is unknown what the prevalence of smokeless tobacco use is in worker groups where there are workplace smoking restrictions. This research brief examined the relationship between occupational status, the presence of workplace smoking restrictions, and smokeless tobacco use among a nationally representative sample of US adults. We found most US workers did not use smokeless tobacco and that prevalence of smokeless tobacco rates was relatively stable throughout the study period. There were large differences in the use of smokeless tobacco across worker groups. Workers employed in occupations where smoking was restricted had lower rates of smokeless tobacco use compared to workers who could smoke at work, with farm and blue collar workers having the highest prevalence. Findings from the present analysis provide strong support the passage of clean indoor air legislation to decrease, not increase, smokeless tobacco use.

### Human Participant Protection

The protocol was approved by the institutional review board of the University of Miami, Miller School of Medicine.

## Competing interests

The authors declare that they have no competing interests.

## Authors' contributions

NAD, DJL, and LEF are responsible for the integrity of the data and the accuracy of the data; WGLB and KLA were responsible for the data analysis; and KEM, EPD, and AJCM worked to conceptualize ideas, interpret findings, and review drafts of the manuscript. All authors have read and approved the final manuscript.
